# High‐mobility group box‐1 promotes vascular calcification in diabetic mice via endoplasmic reticulum stress

**DOI:** 10.1111/jcmm.16075

**Published:** 2021-03-16

**Authors:** Zheng Chen, Ran Li, Li‐Gang Pei, Zhong‐Hai Wei, Jun Xie, Han Wu, Biao Xu

**Affiliations:** ^1^ Department of Cardiology Nanjing Drum Tower Hospital the Affiliated Hospital of Nanjing University Medical School Nanjing China

**Keywords:** diabetes, endoplasmic reticulum stress, high‐mobility group box‐1, vascular calcification

## Abstract

Several studies reported the role of endoplasmic reticulum stress (ERS) in vascular calcification. High‐mobility group box‐1 (HMGB‐1) plays a substantial role in diabetes and its complications. However, relatively little information is available regarding the association between HMGB‐1 and calcification, and the underlying mechanism has still remained elusive. Therefore, in the present study, we attempted to indicate whether HMGB‐1 could promote vascular calcification via ERS in diabetes. After induction of diabetes by Streptozotocin (STZ), mice were treated with glycyrrhizin (Gly) or 4‐phenylbutyrate (4‐PBA). Mineral deposition was confirmed by reverse transcription‐polymerase chain reaction (RT‐PCR) and calcium assay. In cell experiments, calcification of vascular smooth muscle cells (VSMCs) was performed with Alizarin Red staining, alkaline phosphatase (ALP) activity and RT‐PCR. Expression and location of HMGB‐1 in aortic tissue were detected by Western blotting, immunocytochemistry (ICC) and immunohistochemistry (IHC). Diabetic mice demonstrated increased HMGB‐1 expression, ERS and vascular calcification. However, inhibition of HMGB‐1 with Gly or inhibition of ERS with 4‐PBA ameliorated the enhanced vascular calcification and ERS in diabetic mice. In vitro experiments unveiled that inhibition of HMGB‐1 attenuated advanced glycation end products (AGEs)‐induced ERS in VSMCs. In addition, AGEs promoted translocation and secretion of HMGB‐1 in VSMCs, which was reversed by 4‐PBA. Moreover, VSMCs exhibited increased mineralization and osteogenic gene expressions in response to HMGB‐1 and AGEs. However, inhibition of ERS with 4‐PBA partially, although noticeably, attenuated VSMC calcification induced by HMGB‐1. Thus, diabetes induced translocation and secretion of HMGB‐1 via ERS, which resulted in calcification in diabetic mice and in AGEs‐treated VSMCs.

## 1 INTRODUCTION

Cardiovascular complications have been identified as principal cause of morbidity and mortality in patients with diabetes mellitus (DM).^1^ Increasing evidence demonstrates that DM is associated with coronary artery calcification (CAC),^2^, ^3^ which is an independent risk factor for cardiovascular events.^4^, ^5^ DM is deemed as a coronary artery disease (CAD) equivalent, which doubles or even triples the CAD incidence. Despite the fact that the increased prevalence of DM may result in a higher incidence of CAC, therapeutic strategies aiming to effectively prevent or reduce CAC in patients with diabetes have been still rarely reported because of the incomplete understanding of the underlying mechanisms.

High‐mobility group box‐1 (HMGB‐1), a highly conserved nuclear protein, can be translocated into the cytoplasma and released into extracellular space under particular conditions, such as diabetes and inflammation.^6^ HMGB‐1 can bind to at least three distinct receptors, including toll‐like receptor 4 (TLR4), toll‐like receptor 2 (TLR2) and receptor for advanced glycation end products (RAGEs), thereby activating downstream signalling cascades. With regulating inflammation, fibrosis, migration, oxidative stress and apoptosis, HMGB‐1 showed to play a critical role in diabetic cardiovascular diseases.^7^ Advanced glycation end products (AGEs), a diverse group of highly oxidant compounds with pathogenic significance in diabetes and in several other chronic diseases, was associated with CAC in patients with DM.^8^ Our previous in vitro experiments demonstrated that AGEs induced secretion of HMGB‐1 from various cells.^9^, ^10^ Recent studies in patients and animal models reported association between HMGB‐1 and calcific aortic valve disease (CAVD), because tissue and plasma levels of HMGB‐1 were increased in patients with CAVD.^11^, ^12^ Taken together with the previous finding that HMGB‐1 mediates high‐glucose‐induced calcification in vascular smooth muscle cells (VSMCs) of saphenous veins,^13^, ^14^ it has still remained elusive whether HMGB‐1 could link diabetes with vascular calcification.

Endoplasmic reticulum (ER) is a critical organelle that responds to changes in homeostasis, including calcium homeostasis, modification and protein synthesis. When the structure and function of ER is disrupted, a series of cellular responses are made to avoid the accumulation of unfolded proteins in the ER, which is known as endoplasmic reticulum stress (ERS). Several studies have demonstrated that ERS promoted development and progression of chronic kidney disease (CKD)‐induced vascular calcification.^15^, ^16^

It was previously found that HMGB‐1 could promote inflammatory response via RAGE‐mediated stimulation of ERS in endothelial cells.^17^ Recently, HMGB‐1 was shown to be involved in clostridium difficile toxin A‐induced ERS in murine colon adenocarcinoma cells.^18^ Furthermore, ERS induced higher cytoplasmic expression and secretion of HMGB‐1 in tumour‐infiltrating lymphocytes in triple‐negative breast cancer.^19^ Thus, the present study aimed to indicate whether HMGB‐1 could promote vascular calcification via ERS in diabetic mice and VSMCs.

## 2 METHODS

### 2.1 Induction of diabetes

All animal protocols were approved by Animal Care and Use Committee of the Affiliated Drum Tower Hospital of Nanjing University Medical School. C57bl/6 mice were obtained from Model Animal Research Center of Nanjing University. Male mice were randomly assigned into four groups and treated as follows: (a) control group (non‐diabetic mice without Streptozotocin (STZ)/ glycyrrhizin (Gly)/4‐phenylbutyrate (4‐PBA)), (b) STZ group (diabetic mice with STZ injection), (c) STZ + Gly group (STZ‐induced diabetic mice with Gly (Sigma‐Aldrich)) and (d) STZ + 4‐PBA group (STZ‐induced diabetic mice with 4‐PBA (Sigma‐Aldrich)). A model of STZ‐induced diabetic mice was established as previously described.^20^ Briefly, mice were injected with STZ (40 mg/kg; Sigma‐Aldrich) for five consecutive days. When mice were considered as diabetes with blood glucose level > 13.9 mmol/L, some mice were treated with Gly (10 mg/kg/d) or 4‐PBA (50 mg/kg/d) one week after injection of STZ. Bodyweight and blood glucose level were measured every 4 weeks until the animals were killed.

### 2.2 Cell culture and treatment

VSMCs were obtained from Keygen Biotech. The cells were cultured in DMEM containing 10% foetal bovine serum and maintained at 37°C in 5% CO_2_ environment with medium changed every 2 days. For HMGB‐1 induction experiment, the cells were exposed to AGEs (100 μg/mL) for 0, 6, 12 and 24 hours. Then, 100 μg/mL AGEs for 24 hours were used in the following experiments. For the in vitro calcification experiments, VSMCs were incubated with β‐glycerophosphate (β‐GP, 10 mmol/L) in medium containing HMGB‐1 (100, 200, 400 μg/mL) or TM (1, 2, 4 mg/L) for two weeks. Then, 200 μg/mL HMGB‐1 was used in the following experiments. For the inhibitory studies, 10 mmol 4‐PBA or 100 μmol/L Gly was pre‐treated for 2 hours before coincubation with AGEs or HMGB‐1.

### 2.3 Enzyme‐linked immunosorbent assay (ELISA)

Serum level of HMGB‐1 was detected using ELISA according to the manufacturer's protocol (Keygen Biotech Co., Ltd.).

### 2.4 Western blot analysis

Cells and aortic tissues were lysed in ice‐cold lysis buffer (Beyotime Institute of Biotechnology) supplemented with protease inhibitor cocktail and phosphatase inhibitors (Roche Diagnostics; 100 μL per well of a 6‐well plate). Nuclear proteins and cytosolic proteins were extracted using Cytoplasmic and Nuclear Protein Extraction Kit (Beyotime Institute of Biotechnology). Supernatants were harvested and quantified by Pierce bicinchoninic acid (BCA) Protein Assay Kit (Thermo Fisher Scientific). Protein samples were separated by sodium dodecyl sulphate‐polyacrylamide gel electrophoresis (SDS‐PAGE) and electroblotted onto polyvinylidene difluoride (PVDF) membranes (Merck Millipore). After blocking with blocking buffer containing 5% low‐fat milk in phosphate‐buffered saline (PBS) with 0.1% Tween‐20, the membranes were incubated overnight with the following antibodies: β‐actin (Santa Cruz Biotechnology), HMGB‐1 (Bioworld Technology, Inc), glucose‐regulated protein 78 (GRP78) (Santa Cruz Biotechnology), PKR‐like ER kinase (PERK) (Cell Signaling Technology), pPERK (Cell Signaling Technology), inosital‐requiring enzyme 1 (IRE1) (Cell Signaling Technology) and pIRE1 (Cell Signaling Technology). All immunoblots were detected by an enhanced chemiluminescent reagent kit and exposed to X‐ray films. β‐actin was used as a loading control (Abcam). The band intensities were quantified using Quantity One analysis software (Bio‐Rad Laboratories Inc).

### 2.5 Reverse transcription‐polymerase chain reaction (RT‐PCR)

Total RNA was extracted using mRNA isolation kit according to the manufacturer's instructions (Takara). In RT‐PCR, an RNA population was converted into cDNA by reverse transcription (RT), and then, the cDNA was amplified by PCR. The sequences of the primers used were as follows: runt‐related transcription factor 2 (Runx2) (mice), CCAGGCAGGTGCTTCAGAACTG (forward), GGTAGTGAGTGGTGGCGGACAT (reverse); osteocalcin (OCN) (mice), GCTACCTTGGAGCCTCAGTC (forward), ATGCGTTTGTAGGCGGTCTT (reverse); Runx2 (human), CCCAGGCAGTTCCCAAGCATTT(forward), GGTAGTGAGTGGTGGCGGACAT (reverse); OCN (human), GGCAGCGAGGTAGTGAAGAGAC (forward), GGTCAGCCAACTCGTCACAGTC (reverse); bone morphogenetic protein‐2 (BMP‐2) (human), GGTCCTGAGCGAGTTCGAGTTG (forward), TGACCTGAGTGCCTGCGATACA (reverse). Quantitative RT‐PCR (RT‐qPCR) was performed with SYBR green fluorescence (Bio‐Rad Laboratories Inc). β‐actin, a housekeeping gene, was used for internal normalization. Gene expressions were analysed using the 2^–ΔΔCt^ method.

### 2.6 Immunocytochemistry (ICC)

Cells were washed with PBS and fixed with 4% paraformaldehyde (PFA). After incubation with 0.1% Triton X‐100 for 2 minutes on ice, the cells were washed twice with cold PBS. Then, the cells were blocked with bovine serum albumin (BSA) and incubated with HMGB‐1 at 37°C for 30 minutes. After washing with PBS, the cells were incubated with appropriate fluorescently labelled secondary antibodies (Invitrogen) at 30°C in the dark. Finally, 2‐(4‐Amidinophenyl)‐6‐indolecarbamidine dihydrochloride (DAPI, Sigma‐Aldrich) was used to stain the cell nuclear. The cells were visualized under a fluorescence microscope (Olympus).

### 2.7 Immunohistochemistry

Aortic tissues were immediately fixed for 2 hours in 4% methanol‐free formaldehyde. The fixed samples were then embedded into paraffin and were cut into slices. Heat‐mediated antigen retrieval was performed with citrate buffer. After blocking, paraffin‐embedded sections were stained overnight at 4°C with HMGB‐1 antibody and then with corresponding secondary antibodies for 1 hour. Next, 3,3′‐diaminobenzidine (DAB) was used as chromogen, and images were taken using a microscope.

### 2.8 Alizarin Red staining and calcium assay

Mineral deposition was confirmed by Alizarin Red staining and calcium assay according to the manufacturer's instructions. Briefly, the cells were washed thrice with PBS and fixed with glutaraldehyde and then were incubated with Alizarin Red S (Beyotime Institute of Biotechnology) for 10 minutes at room temperature. After washing with PBS, the images of calcium nodules were taken by microscope. Alizarin Red dye was quantified by spectrophotometry and normalized to protein level. After washing with PBS for three times, the samples were decalcified with 0.6 mmol/L HCl at 4°C for 24 hours. Calcium was detected with Arsenazo III calcium measurement kit (StanBio Laboratories Inc) according to the manufacturer's protocol. Protein content was measured using BCA protein assay. The amount of calcium was normalized to total protein and expressed as fold change compared with control.

### 2.9 Alkaline phosphatase (ALP) staining and activity

After the designated treatment, the cells were fixed for 3 minutes and stained with ALP (Nanjing Jiancheng Bio‐Engineering Institute Co. Ltd.) for 15 minutes according to the manufacturer's instructions. Besides, ALP activity and protein concentrations were detected with ALP activity kit and BCA assay kit, respectively. ALP activity was evaluated with normalization to the protein.

### 2.10 Statistical analysis

All experiments were replicated at least three times independently. All data were expressed as mean ± standard error of the mean (SEM). Statistical analysis was conducted with SPSS 21.0 software (IBM). Comparing the results between two groups was carried out using the Student's *t* test, and multiple groups were compared by analysis of variance (ANOVA). *P* < .05 was considered statistically significant.

## 3 RESULTS

### 3.1 Up‐regulated expression of HMGB‐1 in aortas of STZ‐induced diabetic mice

**Figure 1:**
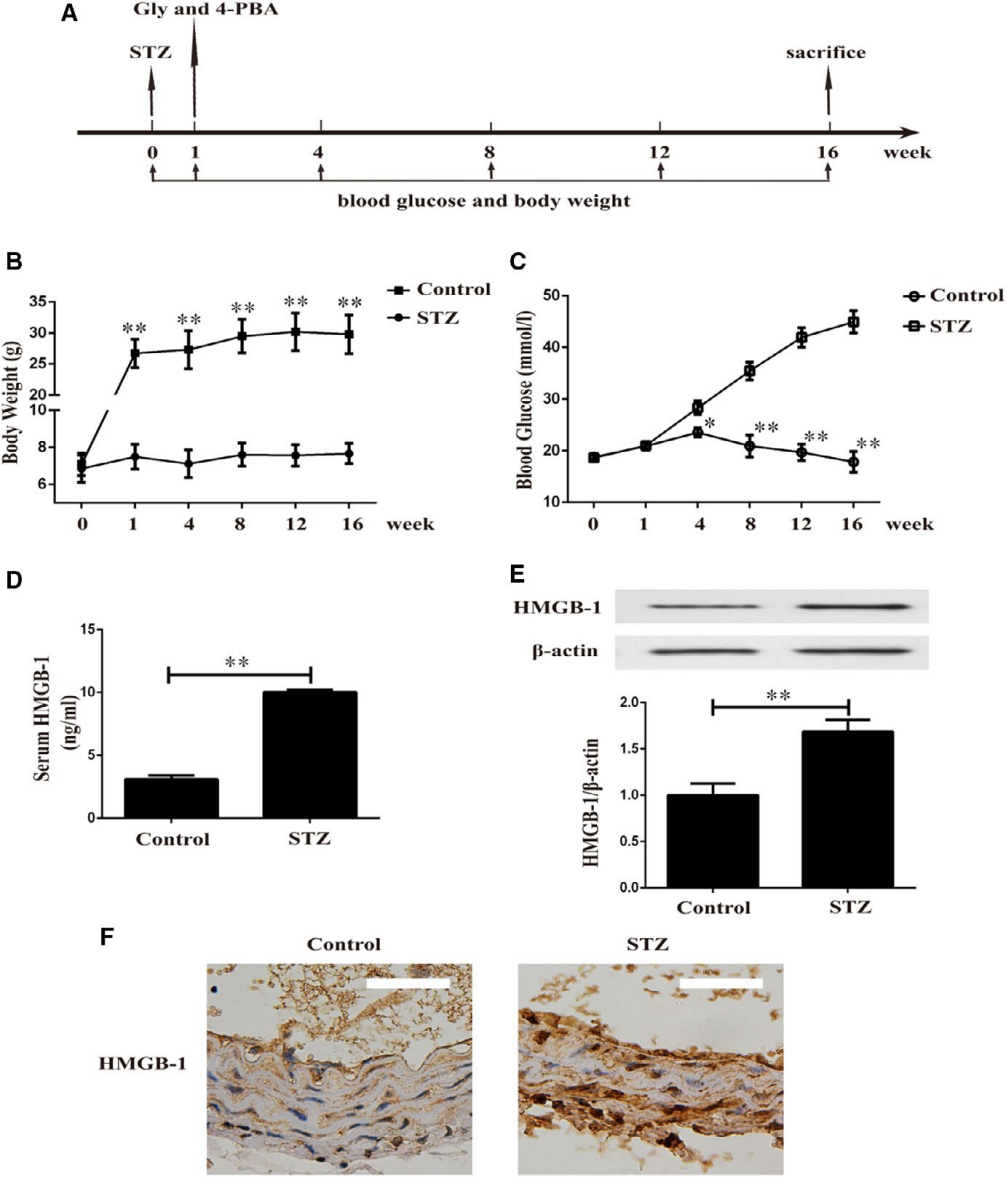
Increased HMGB‐1 expression in aortas of type 1 diabetic mice. A, Protocol of induction of diabetes and experimental design. B, Bodyweight was measured in non‐diabetic and diabetic mice. **P < .01 vs Control group; n = 6. C, Blood glucose level was measured in non‐diabetic and diabetic mice. *P < .05 vs Control group; **P < .01 vs Control group; n = 6. D, Serum HMGB‐1 level in control and STZ groups. **P < .01 between two groups; n = 6. E, Expression of HMGB‐1 in diabetic aortas of two groups. **P < .01 between two groups; n = 8. F, Immunohistochemistry of HMGB‐1 in aortas of non‐diabetic and diabetic mice. Scale bar = 50 um; n = 6. STZ: 40 mg/kg

One week after injection of STZ, diabetic mice were treated with Gly or 4‐PBA for another 16 weeks (Figure 1A). We observed a sustained increase in bodyweight in control group and poor body gain in STZ‐induced diabetic mice (Figure 1B). Blood glucose level was significantly higher in STZ‐induced diabetic mice than non‐diabetic mice during the experiment (Figure 1C). As shown in Figure 1D, diabetic mice exhibited noticeably increased serum HMGB‐1 level. Furthermore, Western blotting (Figure 1E) and IHC (Figure 1F) unveiled up**‐**regulated expression of HMGB‐1 in aortas of STZ‐induced diabetic mice.

### 3.2 Diabetes induced translocation and secretion of HMGB‐1 via ERS in VSMCs

**Figure 2:**
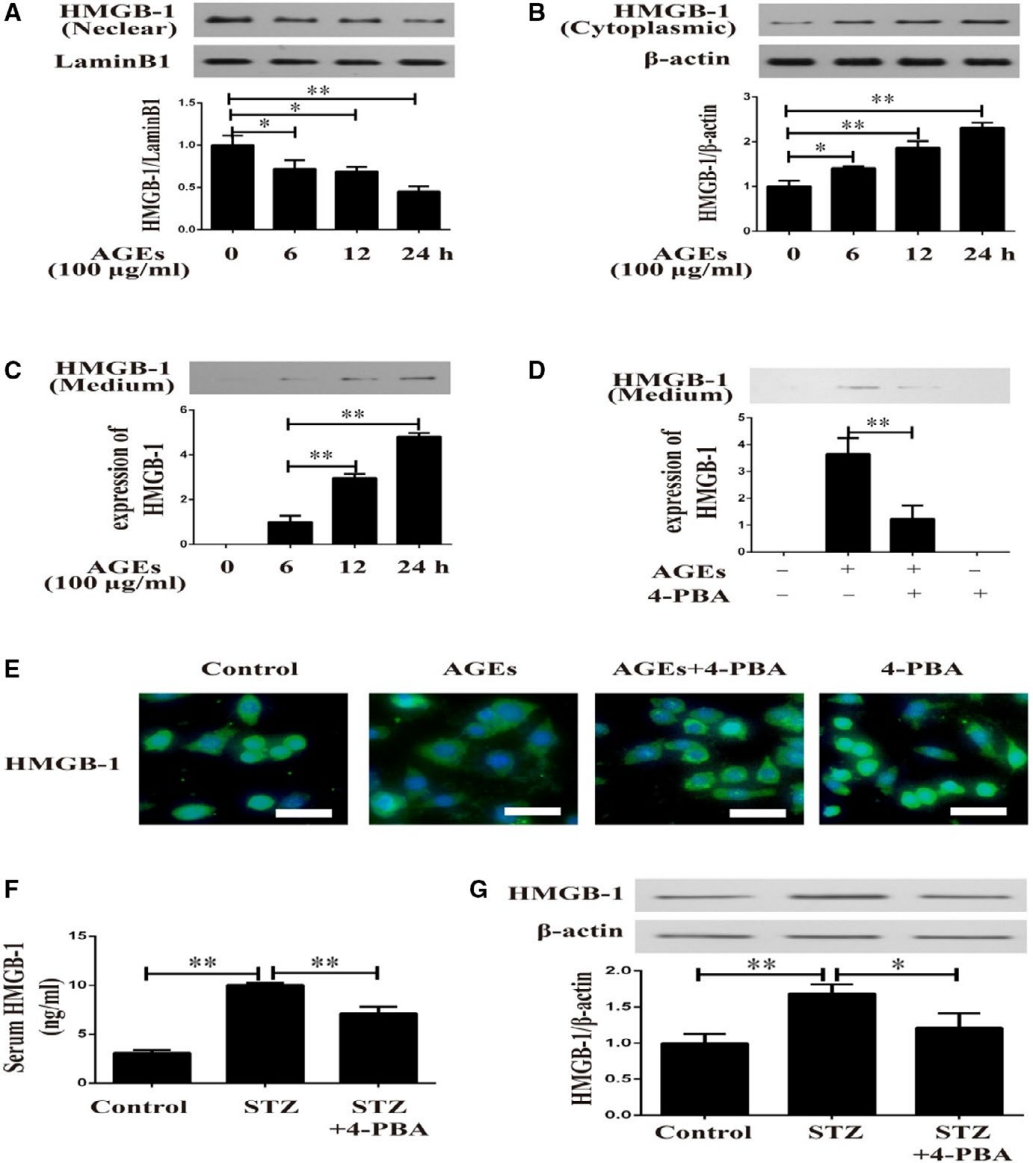
Diabetes‐induced HMGB‐1 secretion was attenuated by 4‐PBA. A, HMGB‐1 expression in nuclear in AGEs‐induced VSMCs in different time‐points. B, Cytoplasmic HMGB‐1 expression in AGEs‐induced VSMCs in different time‐points. C, HMGB‐1 levels in cell supernatant of AGEs‐induced VSMCs in different time‐points. D, Effects of 4‐PBA on AGEs‐induced up‐regulation of HMGB‐1 in VSMCs. E, Effects of 4‐PBA on translocation of HMGB‐1 in AGEs‐induced VSMCs. F, Serum HMGB‐1 levels in the three groups. G, Western blotting showing HMGB‐1 expression in aorta in control, STZ, and STZ + 4‐PBA groups. *P < .05 between the two groups; **P < .01 between the two groups; Scale bar = 50 μm; n = 3. AGEs: 100 μg/mL, 4‐PBA (in vitro): 10 mmol/L, STZ: 40 mg/kg, 4‐PBA (in vivo): 50 mg/kg/d

Herein, in vitro and in vivo experiments were performed to detect the role of ERS in diabetes‐induced HMGB‐1 secretion. In response to AGEs for different time‐points, VSMCs showed decreased HMGB‐1 expression in nuclear (Figure 2A) and increased cytoplasmic HMGB‐1 expression (Figure 2B) in a time‐dependent manner. As illustrated in Figure 2C, AGEs induced up**‐**regulated HMGB‐1 protein levels in conditioned medium of VSMCs. To further understand the underlying mechanism, 4‐PBA (an inhibitor of ERS) was used in the following experiments. 4‐PBA normalized the increased HMGB‐1 level in supernatants of AGEs‐induced VSMCs (Figure 2D). In addition, immunofluorescence analysis indicated that AGEs induced translocation of HMGB‐1 from nuclear to cytoplasm, which was attenuated by 4‐PBA (Figure 2E). To validate this observation in animal models of diabetes, HMGB‐1 expressions in serum and aortas were detected in diabetic mice with 4‐PBA treatment. As displayed in Figure 2F,G, serum HMGB‐1 level and HMGB‐1 expression in aortas were diminished in animal models of diabetes when treated with 4‐PBA.

### 3.3 Diabetes‐induced ERS and vascular calcification were attenuated by Gly or 4‐PBA

**Figure 3:**
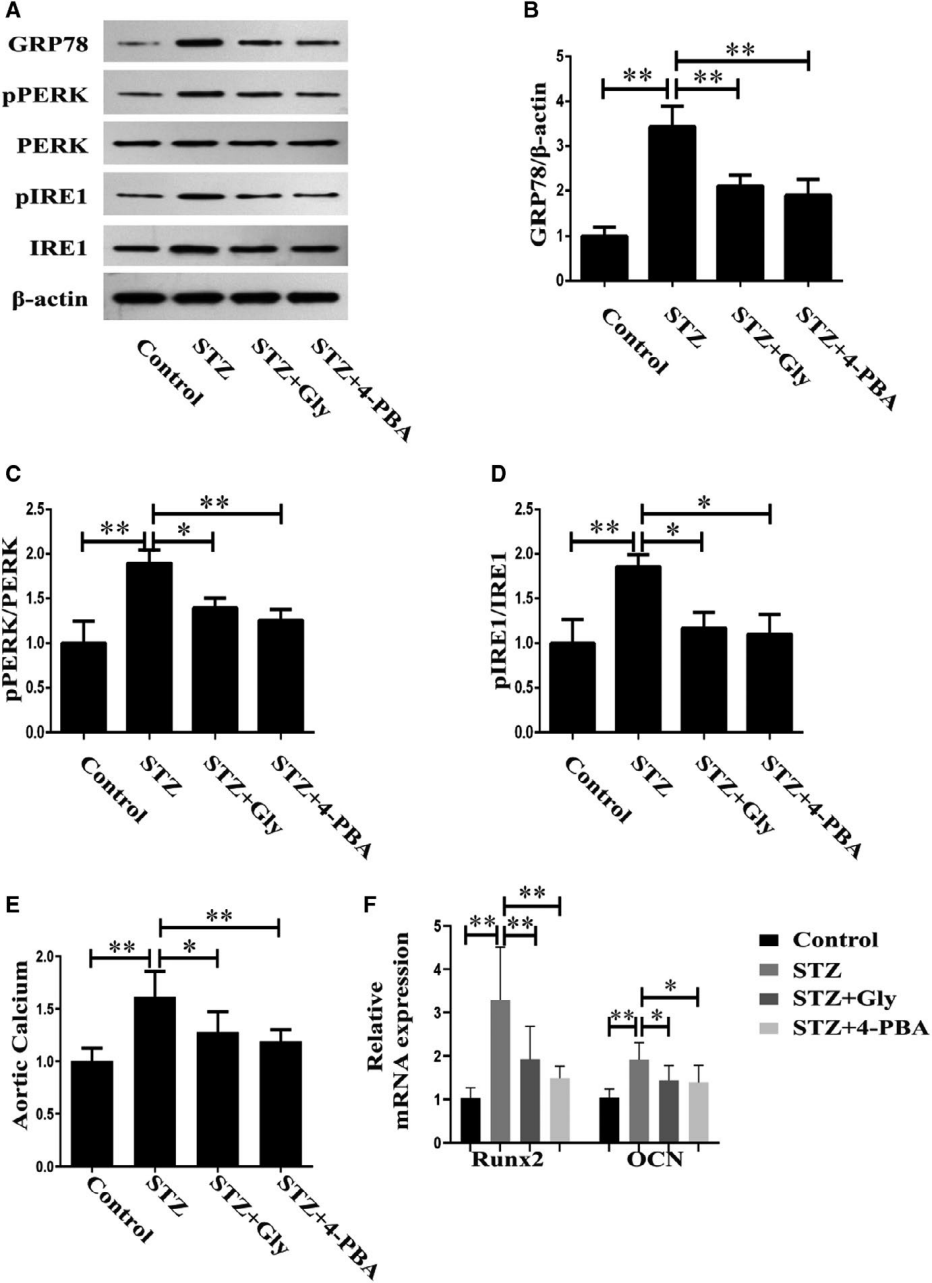
Effects of Gly and 4‐PBA on diabetes‐induced ERS and vascular calcification in diabetic mice. A, Western blotting of ERS‐related proteins in different groups. B, Quantitative analysis of GRP78. C, Quantitative analysis of PERK activity. D, Quantitative analysis of IRE1 activity. E, Aortic calcium levels in different groups. F, mRNA levels of Runx2 and OCN in different groups. *P < .05 between the two groups; **P < .01 between the two groups; n = 6. AGEs: 100 μg/mL, Gly (in vitro): 100 μmol/L, 4‐PBA (in vitro): 10 mmol/L, STZ: 40 mg/kg, Gly (in vivo): 10 mg/kg/d, 4‐PBA (in vivo): 50 mg/kg/d

The next aim of the investigation was to ascertain the roles of HMGB‐1 and ERS in diabetes‐induced vascular calcification in diabetic mice. Firstly, we detected the expression levels of ERS markers in mice models of diabetes treated with Gly or 4‐PBA. As illustrated in Figure 3A‐D, STZ‐induced diabetic mice displayed increased expression of GRP78 in parallel with elevated activities of PERK and IRE1 in aortas, which were inhibited by Gly or 4‐PBA. Secondly, we measured aortic calcium and mRNA of calcification‐related proteins to evaluate vascular calcification. Compared with control group, a significantly increased aortic calcium was noted in STZ‐induced diabetic mice. However, the elevated aortic calcium could be restored by treatment with Gly or 4‐PBA (Figure 3E). RT‐PCR revealed significantly up**‐**regulation of calcification markers in diabetic aortas, including Runx2 and OCN, suggesting that diabetes induced vascular calcification. However, inhibition of HMGB‐1 or ERS reversed the effects of diabetes on vascular calcification, as demonstrated by the findings that these mRNA expressions were attenuated by treatment with Gly or 4‐PBA (Figure 3F).

### 3.4 HMGB‐1 and tunicamycin (TM) induced calcification in VSMCs

**Figure 4:**
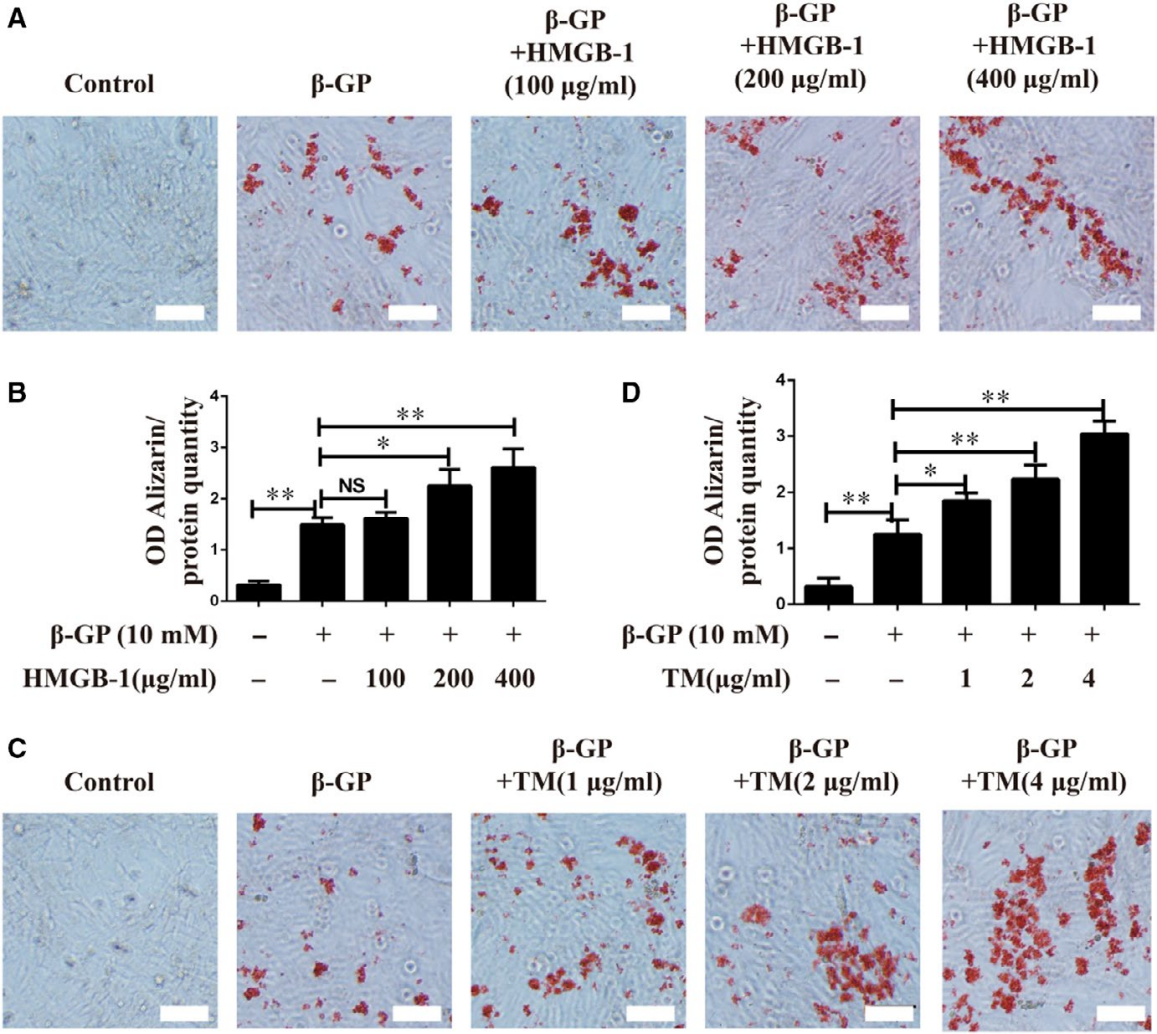
Effects of incubation of VSMCs with HMGB‐1 and TM on calcification. A, Representative images of Alizarin Red staining of VSMCs incubated with HMGB‐1. B, Quantification of Alizarin Red staining of VSMCs incubated with HMGB‐1. C, Representative images of Alizarin Red staining of VSMCs incubated with TM. D, Quantification of Alizarin Red staining of VSMCs incubated with TM. *P < .05 between the two groups; **P < .01 between the two groups; NS, no significance between the two groups; Scale bar = 100 μm; n = 3. β‐GP: 10 mmol/L

As in vivo experiment showed that inhibition of HMGB‐1 or ERS prevented diabetes‐induced vascular calcification, in vitro experiments on VSMCs were performed to confirm the roles of HMGB‐1 and ERS in vascular calcification. VSMCs were cultured with β‐GP in the presence of HMGB‐1 at concentrations ranging from 0 to 400 μg/mL, and treatment with HMGB‐1 at a dose of 200 μg/mL dramatically enhanced calcification, as measured by Alizarin Red staining (Figure 4A,B). In order to indicate whether ERS could contribute to calcification in VSMCs, TM (an ERS inducer) was added into β‐GP‐induced VSMCs. As depicted in Figure 4C,D, TM induced calcification in VSMCs in a dose‐dependent manner, suggesting that induction of ERS promoted calcification in VSMCs.

### 3.5 HMGB‐1 mediated AGEs‐induced ERS and calcification in VSMCs

**Figure 5:**
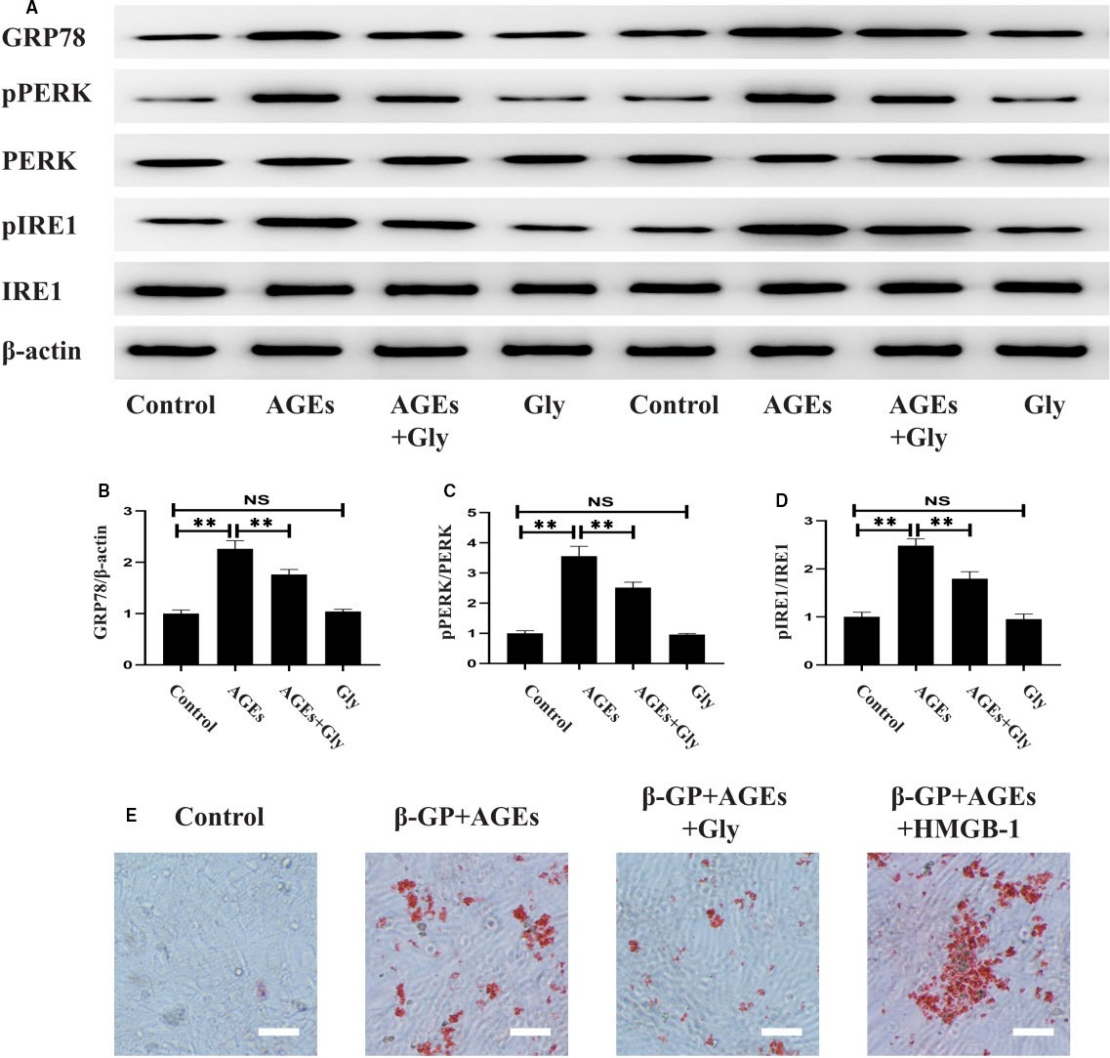
HMGB‐1 promoted AGEs‐induced ERS and calcification in VSMCs. A, Western blotting of ERS‐related proteins in different groups. B, Quantitative analysis of GRP78. C, Quantitative analysis of PERK activity. D, Quantitative analysis of IRE1 activity. E, Representative images of Alizarin Red staining of VSMCs in response to AGEs, HMGB‐1 and 4‐PBA. **P < .01 between the two groups; NS, no significance between the two groups; Scale bar = 100 μm; n = 3. AGEs: 100 μg/mL, Gly: 100 μmol/L, β‐GP:10 mmol/L, HMGB‐1: 200 μg/mL

In line with the results of in vivo experimentz, VSMCs showed increased ERS in response to AGEs challenge, which was reversed by pre‐treatment with Gly (Figure 5A‐D). To confirm the effect of HMGB‐1 in diabetes‐induced calcification, HMGB‐1 and Gly were pre‐treated in AGEs‐induced VSMCs. As shown in Figure 5E, inhibition of HMGB‐1 by Gly significantly prevented AGEs‐induced cell calcification in VSMCs. Furthermore, AGEs‐induced calcification in VSMCs was enhanced by HMGB‐1.

### 3.6 Inhibition of ERS reversed the effects of HMGB‐1‐meidated calcification in VSMCs

**Figure 6:**
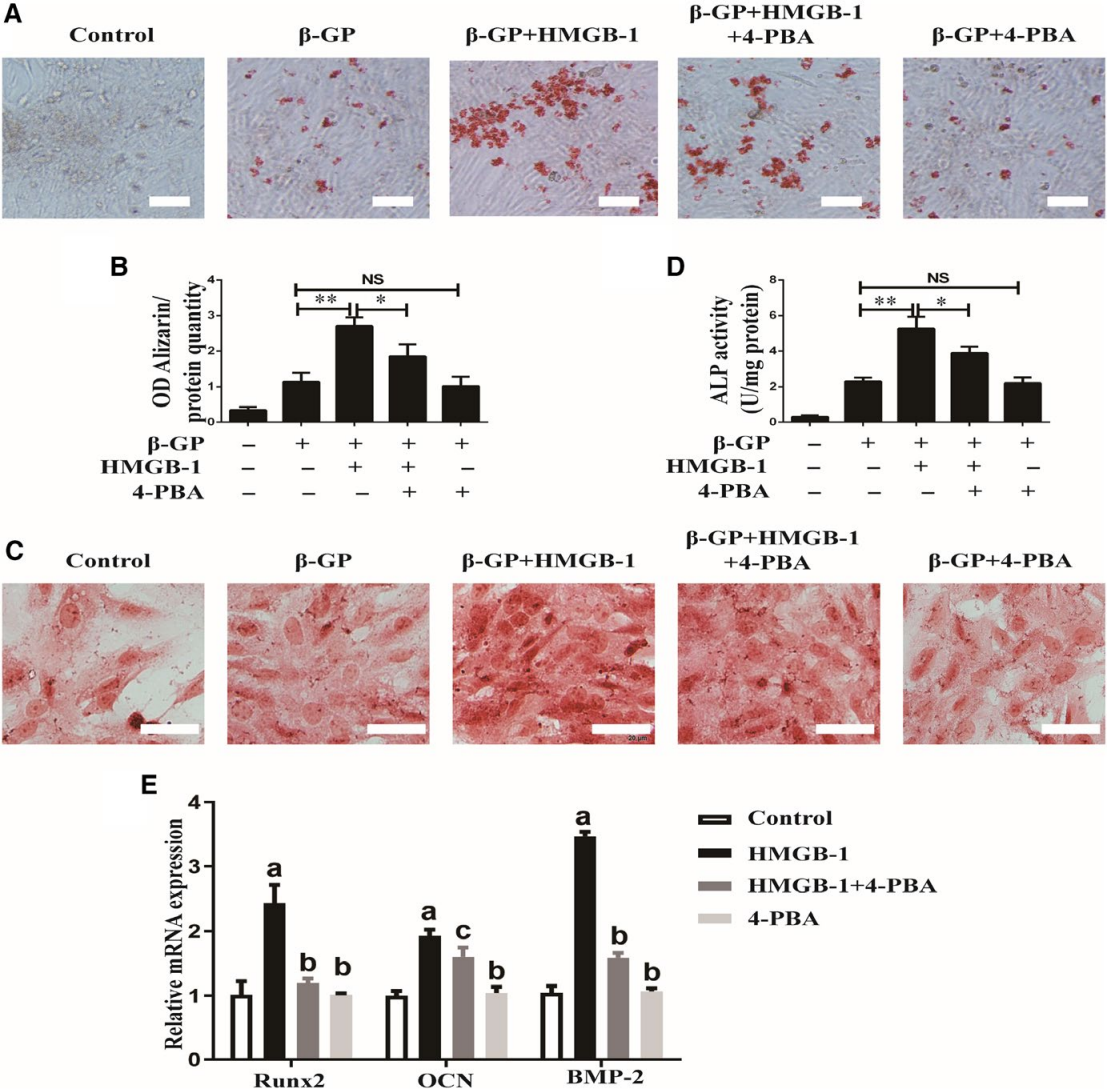
Effects of 4‐PBA on calcification of VSMCs induced by HMGB‐1. A, Representative images of Alizarin Red staining of VSMCs in response to HMGB‐1 and 4‐PBA. n = 3; Scale bar = 100 μm. B, Quantification of Alizarin Red staining of VSMCs incubated with HMGB‐1 and 4‐PBA. *P < .05 between the two groups; **P < .01 between the two groups; NS, no significance between the two groups; Scale bar = 100 μm; n = 3. C, Representative images of ALP staining of VSMCs in response to HMGB‐1 and 4‐PBA. Scale bar = 50 μm; n = 3. D, Quantification of ALP staining of VSMCs incubated with HMGB‐1 and 4‐PBA. *P < .05 between the two groups; **P < .01 between the two groups; NS; no significance between the two groups; n = 3. E, mRNA expressions of Runx2, OCN, and BMP‐2 in VSMCs in response to HMGB‐1 and 4‐PBA. a, *P* < .01 vs Control group; b, *P* < .01 vs HMGB‐1 group; c, *P* < .05 vs HMGB‐1 group; n = 3. β‐GP:10 mmol/L, HMGB‐1:200 μg/mL, 4‐PBA: 10 mmol/L

As both HMGB‐1 and ERS played pivotal roles in diabetes‐induced calcification, we further tested the role of ERS in HMGB‐1‐induced calcification in VSMCs. Alizarin Red staining unveiled that inhibition of ERS by 4‐PBA prevented HMGB‐1‐induced formation of mineralized nodule in VSMCs (Figure 6A,B). Similarly, HMGB‐1‐induced ALP activity was attenuated by 4‐PBA (Figure 6C,D). As shown in Figure 6E, HMGB‐1 induced mRNA expressions of Runx2, OCN and BMP‐2, which were phenotypic markers of calcifying VSMCs. However, treatment with 4‐PBA inhibited these mRNA expressions, suggesting that ERS contributed to HMGB‐1‐induced calcification in VSMCs.

## 4 DISCUSSION

**Figure 7:**
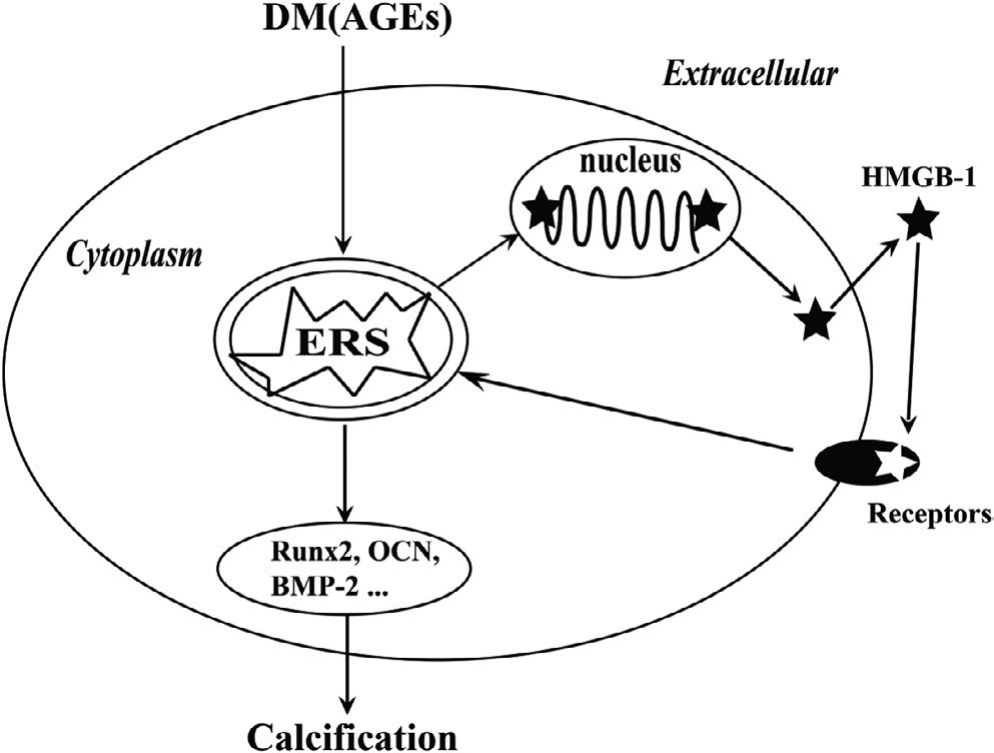
The proposed mechanism in which diabetes induced vascular calcification. AGEs induced secretion of HMGB‐1 via ERS, and extracellular HMGB‐1 increased expression of calcification‐related proteins and ultimately promoted calcification of VSMCs

In summary, the findings of the current study suggested that inhibition of HMGB‐1 and ERS attenuated vascular calcification in STZ‐induced diabetic mice. Additionally, our finding indicated that AGEs induced translocation and secretion of HMGB‐1 in VSMCs via ERS. Eventually, we found that ERS contributed to HMGB‐1‐promoted osteoblastic differentiation in VSMCs. Thus, we assessed the presence of a positive association between HMGB‐1 and ERS in diabetes (Figure 7).

Epidemiological studies show that DM is widely recognized as a modern‐day disease, and vascular calcification is an important complication in diabetic patients.^21^, ^22^ However, the pathogenesis of diabetic vascular calcification needs to be further elucidated. HMGB‐1, a typical damage‐associated molecular pattern protein, exhibited a broad range of biological properties in diabetes and its complications, as discussed in detail in our previous review.^7^ Clinical studies demonstrated that serum HMGB‐1 level was increased in both of type 1^23^ and type 2^24^, ^25^ diabetes mellitus, which reflected pro‐inflammatory state of diabetes. In line with our previous studies,^9^, ^10^ animal experiments in the present study disclosed that serum HMGB‐1 level was noticeably elevated in STZ‐induced diabetic mice. Furthermore, a remarkable increase of HMGB‐1 expression was noted in diabetic aortas compared with controls. Growing evidence illustrated that diabetes up**‐**regulated HMGB‐1 expressions in vascular cells, including endothelial cells and smooth muscle cells.^9^, ^26^, ^27^ The findings were consistent with in vitro results, which showed that AGEs induced HMGB‐1 expressions in VSMCs. Collectively, the above‐mentioned outcome provided the first functional evidence that HMGB‐1 expressions in serum, aorta and vascular cells were up**‐**regulated under diabetic condition.

ERS is defined as accumulation of unfolded or misfolded proteins in the endoplasmic reticulum lumen, resulting in unfolded protein response. ERS can be provoked by various physiological conditions (eg diabetes). Three proximal sensors (ATF6, IRE1 and PERK) of unfolded protein response (UPR) play significant roles in signal transduction events associated with ERS. GRP78 functions as a chaperone by binding to stress sensors in all folding stages. However, ERS is excessively activated when GRP78 is dissociated from ER stress sensors.^28^ The results of the present study indicated that the expressions of GRP78, pPERK/PERK and pIRE1/IRE1 were increased in aortas of STZ‐induced diabetic mice, suggesting enhanced ERS in diabetic aortas. In vitro experiment confirmed this result showing that AGEs up**‐**regulated ERS‐related proteins in VSMCs. However, inhibition of ERS attenuated the expressions of HMGB‐1 in serum and aortas. Thus, it can be concluded that diabetes may promote expression and secretion of HMGB‐1 via ERS.

HMGB‐1 is typically expressed in cell nuclear; however, it may translocate into cytoplasm and be released outside of cells under stress conditions. Emerging evidence demonstrated that HMGB‐1 could be released from VSMCs by different stimulators, such as lipopolysaccharides^29^ and interferon (IFN)‐gamma.^30^ We have previously reported that AGEs induced secretion of HMGB‐1 in endothelial progenitor cells and macrophages via oxidative stress.^9^, ^31^ In the present research, using VSMCs in culture, we further demonstrated that AGEs promoted translocation of HMGB‐1 from nuclear into cytoplasm. Additionally, inhibition of ERS by 4‐PBA partially, although significantly, inhibited AGEs‐induced up**‐**regulation of intracellular HMGB‐1 protein and HMGB‐1 in conditioned medium. A previous study demonstrated that ERS induced secretion of high‐mobility group proteins in triple‐negative breast cancer.^19^ These data lent additional evidence to the hypothesis that diabetes induced secretion of HMGB‐1 from VSMCs via ERS‐dependent manner.

Despite extensive studies concentrated on the role of HMGB‐1 in diabetic complications, there is little information regarding the relationship between HMGB‐1 and vascular calcification in diabetes. A clinical investigation demonstrated that serum HMGB‐1 level was associated with peripheral artery disease in DM patients,^32^ whereas the underlying mechanism has still remained elusive. As described above, diabetic mice and AGEs‐induced VSMCs exhibited increased HMGB‐1 level and calcification. However, treatment with Gly (an inhibitor of HMGB‐1) attenuated vascular calcification in diabetic mice. Furthermore, inhibition of HMGB‐1 reversed cell calcification induced by AGEs in in vitro experiments. Jin et al demonstrated that HMGB‐1 promoted aortic calcification in chronic kidney disease.^33^ In addition, HMGB‐1 induced osteoblastic differentiation and calcification in human aortic valve interstitial cells.^11^, ^34^, ^35^ In our study, HMGB‐1 not only induced calcification but also synergistically promoted AGEs‐induced calcification in VSMCs. Therefore, taken together with the previous findings, it can be concluded that HMGB‐1 may contribute to diabetes‐induced vascular calcification.

In recent years, studying the role of ERS in vascular calcification has significantly attracted scholars’ attention. It was reported that CKD induced by 5/6 nephrectomy activated the ERS response and vascular calcification in apolipoprotein E‐deficient (ApoE^−/−^) mice,^15^ which was inhibited by simvastatin plus ezetimibe.^16^ These findings were consistent with a recent in vitro experiment, which revealed that calcifying media up**‐**regulated expressions of ERS‐related proteins and induced mineralization of VSMCs.^36^ The present study demonstrated that ERS was enhanced in diabetic aortas, as demonstrated by increased expression of GRP78 and activation of PERK and IRE1. In cellular experiment, induction of ERS by TM triggered osteoblastic differentiation of VSMCs. However, inhibition of ERS with 4‐PBA remarkably prevented diabetes‐induced vascular calcification. In favour of this deduction, Hao et al recently found that TM promoted and 4‐PBA inhibited VSMC calcification and phenotype transformation.^37^ Furthermore, high‐glucose‐induced osteoblastic differentiation of VSMCs was markedly suppressed by pre‐treatment with 4‐PBA.^38^ Therefore, ERS may contribute to diabetes‐induced vascular calcification.

We further assessed the mechanistic action of HMGB‐1/ERS on vascular calcification at cellular and molecular levels. As demonstrated in the current study, HMGB‐1 facilitated mineral deposition in VSMCs in a dose‐dependent manner, and inhibition of HMGB‐1 attenuated AGEs‐induced calcification in VSMCs. In line with our results, an earlier report showed that knockdown of HMGB‐1 reduced high Pi‐induced aortic calcification in CKD mice.^33^ Recently, Wang et al found that deficiency of RAGEs alleviated aortic valve calcification via inhibition of ERS.^34^ Therefore, it can be concluded that HMGB‐1 accelerates vascular calcification by regulating ERS.

In summary, the results of the present study indicated that diabetes induced HMGB‐1 secretion via ERS; in turn, HMGB‐1 induced vascular calcification via excessive activation of ERS. Thus, HMGB‐1 plays a substantial role in diabetic vascular calcification via a positive feedback loop involving ERS‐mediated pathway.

## CONFLICT OF INTEREST

The authors declare that there is no conflict of interest.

## AUTHOR CONTRIBUTION

**Zheng Chen:** Project administration (equal). **Ran Li:** Methodology (equal); Project administration (equal). **Gang Li Pei:** Investigation (equal); Methodology (equal). **Hai Zhong Wei:** Formal analysis (equal); Software (equal). **Jun Xie:** Writing‐review & editing (equal). **Han Wu:** Project administration (equal); Writing‐original draft (equal); Writing‐review & editing (equal). **Biao Xu:** Writing‐review & editing (equal).

## Data Availability

All data used during the study appear in the submitted article.
